# Editorial: Perceptual Grouping—The State of The Art

**DOI:** 10.3389/fpsyg.2017.00067

**Published:** 2017-01-30

**Authors:** Birgitta Dresp-Langley, Stephen Grossberg, Adam Reeves

**Affiliations:** ^1^ICube UMR 7357 Centre National de la Recherche Scientifique, Strasbourg UniversityStrasbourg, France; ^2^Graduate Program in Cognitive and Neural Systems, Department of Mathematics, Boston UniversityBoston, MA, USA; ^3^Psychology Department, Northeastern UniversityBoston, MA, USA

**Keywords:** perceptual grouping, boundary formation, figure-ground, depth perception, meaning in life, adaptive behavior

Perceptual neuroscience has identified mechanisms of *perceptual grouping* which account for the ways in which visual sensitivity to ordered structure and regularities expresses itself, in behavior and in the brain. The need to actively construct order, notably representations of objects in depth, is mandated as soon as visual signals reach the retina, given the occlusion of retinal signals by retinal veins and other retinal elements or blur. Multiple stages of neural processing transform fragmented signals into visual key representations of 3D scenes that can be used to control effective behaviors. Since our survival depends on our ability to pick up order in the physical world, and since we conceive the physical world as an ordered one, our perception must somehow be sensitive to order. The articles from this collection illustrate how texture dissimilarity, boundary completion, surface filling-in, and figure-ground segregation generate perceptual order. Structural regularities are required for a visual texture to be discriminated from random noise by human observers, as shown in Katkov et al., who compare the performance of human observers to those of an Ideal Observer and an Order Observer model. The Order Observer model, based on the capture of statistical regularities in textures, is shown to closely reflect the sensitivity of the human visual system. Spatiotemporal boundary formation is critical to the perception of shape boundaries and global motion, as shown here by Erlikhman and Kellman, who demonstrate boundary integration from spatially and temporally sparse transformations of texture. The visual system uses positions and times of element transformations to extract local structural regularities such as edge fragments, which then connect by interpolation processes to produce larger shapes with a spatiotemporal integration constraint of ~80 ms between elements. Structural ambiguity and uncertainty pose a specific problem here. *Perceptual grouping* not only accounts for the ways in which physically defined order is processed by the visual system, but also how such order may be inferred in cases of uncertainty, and how this may give rise to the perception of “illusory” or “phantom” shapes. Roncato et al. discuss a new phenomenon in which illusory streaks irradiate from the vertexes of angular contours, connecting pairs of figures, and forming phantom net shapes that are only clearly visible in a specific figural context, as discussed here in the light of several theories of object contour detection. The real world conditions or configurative order to which *perceptual grouping* is sensitive also determine our aesthetic perception, as shown already elsewhere in a large body of previous work on aesthetic preferences for structural regularities. Here, Pinna et al. discuss a novel variant of *perceptual grouping* called *accentuation for perceptual coupling*. New conditions for grouping that cannot be explained in terms of the classical Gestalt principles (similarity etc.) are shown. This new work reveals the supremacy of dissimilarity as an important figural complement to similarity in the genesis of aesthetic structure, involving novel dynamics of perceptual grouping they call *coupling*. The visual system organizes incoming information early on and interprets a 2D image in terms of the real world, producing configurative order that provides perceptual continuity and enables object-based attention. Grossberg reviews the Form-And-Color-And-DEpth (FACADE) model and its laminar cortical embodiment and extension by the 3D LAMINART models. These have explained, simulated, and predicted many perceptual and neurobiological data about how the visual cortex carries out 3D vision and figure-ground perception, and how these cortical mechanisms generate 3D percepts from 2D pictures of occluding and occluded objects. The models provide a unified explanation of neurophysiological data about cortical area V2 cells reported in a remarkable series of articles from the von der Heydt laboratory. Properties of border ownership, contrast preference, binocular stereoscopic information, selectivity to side-of-figure, Gestalt rules, strength of their modulation by attention, and temporal properties are explained. The segregation of image parts into foreground and background requires border ownership assignment by the visual system and recordings from monkey visual cortex show that many neurons, especially in area V2, are selective to border ownership. These neurons are edge selective and have classical receptive fields (CRF). Their responses are modulated (enhanced or suppressed) depending on the location of a “figure” relative to the edge in their receptive field. Each neuron has a fixed preference for location on one side or the other. This selectivity is derived from image context far beyond the CRF. von der Heydt reviews evidence indicating that border ownership selectivity reflects the formation of early object representations, which he terms “proto-objects.” The evidence includes experiments showing (1) reversal of border ownership signals with change of perceived object structure, (2) border ownership specific enhancement of responses in object-based selective attention, (3) persistence of border ownership signals in accordance with continuity of object perception, and (4) remapping of border ownership signals across saccades and object movements. Grouping, persistence, and remapping are shown to be manifest in low-level visual areas, which challenges some of the traditional views and top-down-only models of *perceptual grouping*. To generate perceptual judgments relative to figure and ground, polarity-invariant and polarity-specific object properties are used by the visual brain. Dresp-Langley and Grossberg demonstrate conditions under which figure-ground is determined by the orientation of contrasts, not by their relative sign. Psychophysical data from human observers responding to structurally ambiguous, fragmented 2D shapes with varying local contrast signs reveal polarity-invariant perceptual grouping predicted by the earliest version of the FACADE theory and, more recently, by the 3D LAMINART models. Research into processes of *perceptual grouping* has important societal and technological aspects, for visual interface design and, in particular, image-guided surgery platforms and simulators. Such implications, brought forward here by Dresp-Langley illustrates why boundary completion and figure-ground segregation are not only essential for realistic scene perception, but also for visually guided action planning and behavior.

## Author contributions

BD wrote the draft of this Editorial. AR and SG made comments and suggested minor amendments.

### Conflict of interest statement

The authors declare that the research was conducted in the absence of any commercial or financial relationships that could be construed as a potential conflict of interest.

